# Determinants of hand hygiene compliance among nurses in US hospitals: A formative research study

**DOI:** 10.1371/journal.pone.0230573

**Published:** 2020-04-07

**Authors:** Madeline Sands, Robert Aunger

**Affiliations:** 1 Department of Infectious Disease, London School of Hygiene and Tropical Medicine, London, United Kingdom; 2 University of Arizona College of Medicine, Tucson, Arizona, United States of America; University of New South Wales, AUSTRALIA

## Abstract

Hand hygiene is the simplest and most effective measure for preventing healthcare-associated infections. Despite the simplicity of this procedure and advances made in infection control, hospital health care workers’ compliance to hand hygiene recommendations is generally low. Nurses have the most frequent patient care interactions, and thus more opportunities to practice hand hygiene. As such, it is important to identify and understand determinants of nurses’ reported compliance. Formative research was undertaken to assess the potential impact of several unexamined factors that could influence HH among nurses: professional role and status, social affiliation, social norms, and physical modifications to the work environment (as well as institutional factors like safety climate). A survey questionnaire was developed primarily to inform the creation of a behaviour change intervention. The survey looked at how these factors influence HH among nurses and sought to identify barriers and levers to reported hand hygiene. It was administered to a survey panel of acute care nurses, working in US hospitals, with a year or more of experience. Multivariate regression modelling suggested that reported hand hygiene compliance was most likely to be a function of a hospital management’s communication openness, perceived performance by peers, increased interactions with patients and other staff members, and the reduction in stress, busyness, and cognitive load associated with role performance. A powerful, effective intervention on HH among nurses therefore could be directed at improving communication openness, consider the impact of perceived performance by peers, increase interactions with patients and staff, and determine how to reduce the stress and cognitive load associated with role performance.

## Introduction

Hand hygiene (HH) is the simplest and most effective measure for preventing healthcare-associated infections (HAIs).[1] Despite the simplicity of this procedure and advances made in infection control, hospital health care workers’ compliance to HH recommendations is generally low.[2] Nurses have the most frequent patient care interactions, and thus more opportunities to practice HH.[3] As such, it is important to identify and understand determinants of nurses’ reported compliance.

Hand hygiene is a complex behaviour with a myriad of motivators and barriers.[1, 4] While the basic behavioural aspects surrounding HH practices in hospital settings have been widely researched, there remain gaps in the literature regarding effective psychological promotion of hand hygiene compliance (HHC).[4] Psychological frameworks have been shown to lead to behaviour change in a wide variety of contexts, especially in the behaviour of healthcare workers (HCWs).[5] Therefore, focusing on determinants of behaviour change and employing psychological behaviour change models can better inform HH improvement strategies.

Behaviour Centred Design (BCD) is a general approach to behaviour change that offers both a Theory of Change for behaviours in addition to a practical process for designing and evaluating interventions.[6] The BCD’s Theory of Change incorporates concepts such as reinforcement learning theory,[7] the evolution of behavioural control,[8] the evolved structure of human motivation,[9] and behaviour settings theory.[10,6] The behaviour settings theory explains the relationship between individuals and the environment—both physical and social.[10] Behaviour is a function of the setting within which it takes place. As such, behaviour settings are situations where people have learned what to expect from the environment and from other people’s behaviours. Each setting has a purpose, a designated place, a set of objects, and a prescribed set of behaviours. Therefore, each person entering a setting expects others, who are also participants, to perform their designated roles.

BCD is associated with a checklist of factors that determine human behaviour, which can be used to direct empirical investigations prior to the design of public health interventions. This checklist includes environmental determinants such as the props and infrastructure that support performance of the behaviour, as well as the psychological characteristics and personal traits required.

The aim of this study is to use the BCD approach to identify determinants that impact the HHC of nurses in intensive and acute care hospital units. A combination of literature review and formative research are used to identify prospective strategies for a behaviour change intervention. Recognizing what motivates and hinders a nurse from practicing HH should aid in the development of successful strategies seeking to improve nurses’ HHC.

### Background

Given the complexity of institutional settings for behaviour change, our data gathering strategy focussed on potentially important factors that have not yet been found to be significant. The literature search began with a background search to develop an understanding for the breadth of the body of literature. The iterative search process became more refined and developed as the review progressed. Once the volume and general scope of the HH field had been determined, parameters were set and search strings were developed [S1 File]. Search strings were developed for concepts encompassing behaviour change, hand hygiene compliance, healthcare workers, healthcare-associated infections, hand hygiene, and interventions. Medline, Web of Science, CINAHL, and Google Scholar databases were electronically searched selecting only for papers written in English. A total of 187 publications were identified this way; after filtering for papers published from January 2002- January 2015, there were 89 papers left to be reviewed. Additional papers and grey literature were identified by searching the references lists of the retrieved papers. We used the WHO’s tables of factors (WHO Table I.2.1) as well as hand hygiene improvement interventions (WHO Table I.2.2) as a framework.[1]

### Categorizing and identifying modifiable factors using BCD

The BCD Checklist itemises all the types of behavioural determinants identified by the BCD approach. Placing the factors from the literature known to influence HHC (Table 1) into the BCD Checklist enables us to see what categories of factors have potential for deeper investigation and could serve as the foundation for further research into HHC (see Table 2). This analysis shows that only a few of these categories have been investigated by intervention-based studies in the literature, and it is apparent that whole categories of factors have not yet been examined by the public health community. Types of potential factors that have been completely ignored thus far are listed without entries in Table 1. It should be noted that even some categories with entries below have not been fully investigated; additional factors could be identified and explored. If we restrict our attention to categories—either with or without entries—which can be readily changed by the types of mechanisms that are both acceptable and within the budget of an average hospital administration, we arrive at the following list of five categories: (1) *motivational psychology*, (2) *reactive psychology* (i.e. habit formation), (3) *modification of the relevant behaviour setting stage*, (4) *role change*, and (*5) social norm manipulation*. These categories will be the focus of this formative research.

**Table 1:** Factors and behaviour change strategies examined in the academic literature.

CATEGORY	SUB-CATEGORY	FACTORS IN THE LITERATURE	BEHAVIOR CHANGE STRATEGIES IN THE LITERATURE
**Brains**	**Executive**	*Identity*: doctor, nurse, nurse assistant *Knowledge/Belief*: lack of knowledge of hand hygiene recommendations, disagreement with regulations, scepticism about efficacy of hand hygiene	Emphasize self-protection Provide knowledge of hand hygiene techniques/ regulations Feedback on performance
	**Motivate**	Fear of ‘dirt’	––
	**Reactive**	––	––
**Body**	**Traits**	Male	––
	**Physiology**	Hand hygiene agent, such as alcohol-based hand rub, causes irritation/dryness	––
	**Senses**	––	––
**Behaviour Setting**	**Stage**	––	––
	**Roles**	Relationship with patient/patient needs Lack of others as role models	––
	**Routine**	High number of hand hygiene opportunities Too busy	Reminders
	**Script**	Forgetfulness Distraction/ Interruption Discretionary refusal	––
	**Norms**	––	––
	**Props**	Automated sink Sink location Lack of soap Wearing gloves Dispensers conveniently located	Improved access to ABHR
**Environment**	**Physical**	––	––
	**Biological**	Activities with high/low risk of cross-contamination	––
	**Social**	Work in intensive care or acute care settings Understaffing	Social influence
**Context**	**Programmatic**	––	––
	**Political**	Lack of institutional priority for hand hygiene compliance Lack of sanctions for non-compliance Lack of safety climate	––
	**Economic**	––	––
	**Social**	––	––

**Table 2:** Characteristics of survey participants.

Variable	N Response (out of 540)	Percent (%)
**Gender**		
Female	490	90.74
Male	50	9.26
**Geographic Location in the United States**		
New England	27	5.00
Middle Atlantic	75	13.89
East North Central	102	18.89
West North Central	43	7.96
South Atlantic	88	16.29
East South Central	24	4.44
West South Central	44	8.15
Mountain	54	10.0
Pacific	83	15.37
**Age**		
20–29 y	46	8.52
30–39 y	124	22.96
40–49 y	104	19.26
50–59 y	183	33.89
≥ 60-69y	83	15.37
**Professional Status**		
Staff nurse	467	86.48
Nurse manager	10	1.85
Assistant nurse manager	13	2.41
Nursing director	3	0.56
Advanced practice nurse	28	5.19
Other	19	3.52
**Medical Specialty**		
Medical/surgical unit (Med/surg)	129	23.89
Intensive care unit (ICU)	108	20.00
Cardiac unit	51	9.44
Emergency	105	19.44
Other (NICU, PACU, Radiology, Oncology, Obstetric)	147	27.22
**Hospital Type**		
Teaching	305	56.48
Non-Teaching	235	43.52
Urban	407	75.37
Rural	133	24.63
System-affiliated	425	78.70
Independent	115	21.30
**Hours Worked Per Week**		
30–35 h	62	11.48
36–40 h	411	76.11
41–45 h	22	4.07
46–50 h	35	6.48
≥ 51–65 h	10	1.85

### Importance of this formative research

Formative research is a critical step in the development of health behaviour change interventions.[6, 11] The purpose of formative research is to assess individuals’ beliefs, perceptions, behaviours, and the structure of the environment itself that may help or hinder program effectiveness. Typically, such research involves significant fieldwork in the relevant context. In the case of this study, the ability of the research team to obtain a comprehensive view of the factors associated with HHC was limited by the logistics of access to hospitals. It was neither possible to take nurses from the floor during their shift nor to engage in substantial observation of their practices without introducing bias into the data collection. Further, there is considerable variation and organization-specific intricacies when it comes to the institutional contexts of HHC, which needs to be understood and considered when creating interventions intended to be widely used. Thus, the decision was made to administer a survey to a large number of nurses with a range variety of experiences across the United States, gaining in breadth what was lacking in terms of depth in the investigation. This survey sought to assess the behavioural change potential of the factors identified by the analysis above.

## Methods

Ethics approval was attained from the London School of Hygiene and Tropical Medicine’s Observational and Interventions Research Ethics Committee (reference number is 14411).

### Sampling procedure

An anonymous internet-based cross-sectional survey was administered between November to December 2015 by a global online sampling and digital data collection company called Dynata—formerly known as Research Now—to a survey panel of acute care nurses, working in various types of hospitals that are geographically distributed across the US, with at least a year or more of experience. There were 19,969 hospital nurses available to take the survey. With a confidence interval of 95% and a margin of error 5%, we calculated that we need a minimum of 377 completed surveys. Dynata screened and recruited participants, and it used an incentive scale based on set time increments. Incentive options allowed panellists to redeem from a range of gift cards, charitable contributions, and other products or services upon completing the survey.

### Survey design

**Fig 1:**
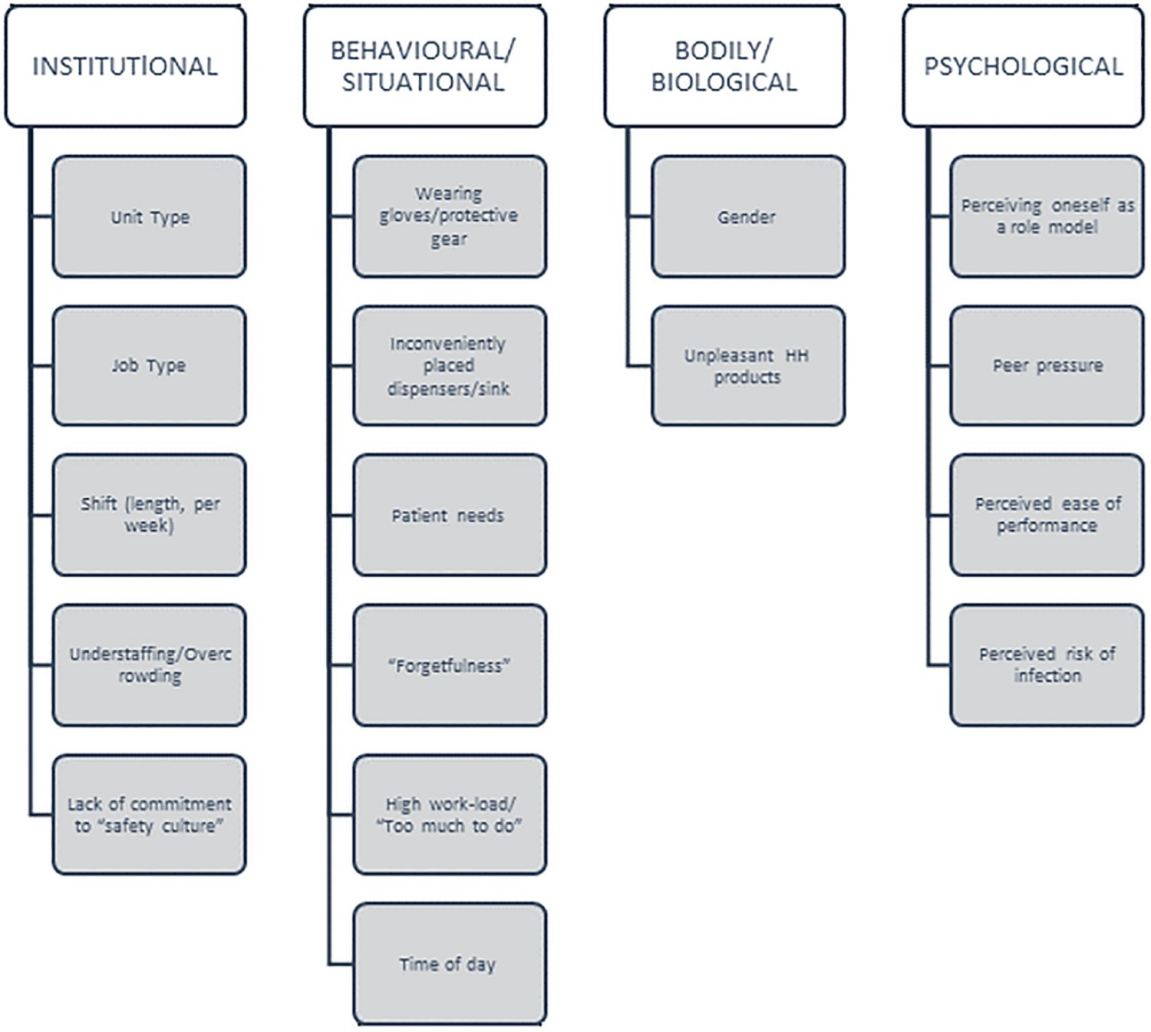
Known hand hygiene compliance factors. To make a more succinct and pertinent list of risk factors for this research project, we first determined which factors have already been found to have a significant impact on HH behaviour from a literature search and review.

The survey concentrates on the five unexamined but modifiable factors that are potential determinants of HHC: (1) motivation, (2) habit, (3) roles, (4) behaviour setting stage, and (5) norms. The survey questions, which draw upon various concepts and measurement tools from fields such as sociology and psychology, are designed to measure the degree to which these factors influence reported HHC [S2 File]. In doing so, a novel questionnaire was developed using techniques—such as vignettes and the self-reported habit index (SRHI)[12]—that have not been commonly or consistently used in HH questionnaires before. The movement of the respondent through the survey is depicted in Fig 1. The explanation of the theoretical underpinnings of the survey with their respective survey questions follow.

#### Role

The role of the nurses was explored through professional identity. Identities are the traits and characteristics, social relations, roles, and social group memberships that define an individual.[13] A professional identity is the sense of self that is associated with the enactment of a professional role.[14, 15] This identity gives members of a profession a definition of self-in-role and the goals, values, norms, and interaction patterns that are associated with their job.[16] This definition of identity is critical to how professionals interpret and behave in various work situations, with identity being both a product of situations and a determinant of behaviour within situations.[13,17] Identity is (a) predicted to influence what individuals are motivated to do, (b) encompasses how individuals think and makes sense of themselves and others, (c) the actions the individuals take, and (d) the individuals’ feelings and abilities to control or regulate themselves.[18, 19]

By learning what qualities, skills, and traits nurses value, the perceived responsibilities of the professional role can be modified to include the responsibility of practicing HH. There is potential for hand hygiene to be integrated into the qualities that nurses perceive to be what a “good” or “ideal” nurse possesses. Respondents are therefore asked to choose five qualities or traits they wish they had exhibited more of during their most recent shift. The following qualities and traits were identified from the literature:[20–27]

$$\begin{array}{ll} \text{Empathy} & \text{Reliability}\\ \text{Respect} & \text{Awareness}\\ \text{Confidence} & \text{Critical}\;\text{Thinking}\\ \text{Technical}\;\text{Competence} & \text{Stress}\;\text{Management}\\ \text{Leadership} & \text{Flexibility}\\ \text{Good}\;\text{Communication}\;\text{Skills} & \text{Physical}\;\text{Endurance}\\ \text{Mental}\;\text{Endurance} & \text{Patient}\;\text{Advocate}\\ \text{Friendliness} & \text{Resourcefulness}\\ \text{Patience} & \text{Responsiveness}\\ \text{Good}\;\text{judgment} & \text{Cooperativeness} \end{array}$$

Respondents were then asked to choose five statements they would least like to hear said about them as a nurse. The statements address undesirable qualities and traits or unfavourable working conditions identified in the literature.[20–27]

$$\begin{array}{l} \text{“I do not provide emotional support to my patients.”}\\ \text{“I am unsure of myself as a nurse.”}\\ \text{“I do not handle stress well.”}\\ \text{“I am not as technically skilled as I should be.”}\\ \text{“I am curt and short with the patients.”}\\ \text{“I do not show leadership qualities.”}\\ \text{“I do not communicate well with others.”}\\ \text{“I neglected a patient.”}\\ \text{“I am not dependable.”}\\ \text{“I am not always aware of what is going on around me.”}\\ \text{“I hurt a patient.”}\\ \text{“I neglected a patient.”}\\ \text{“I do not know my patient’s wants or needs.”}\\ \text{“I am not flexible and able to adapt.”}\\ \text{“I am not a team player.”} \end{array}$$

#### Norms

A social norm is a rule of behaviour that individuals conform to conditionally based on the beliefs that (a) most people in their relevant network conform to this behaviour (this is referred to as an *empirical expectation*), (b) they themselves believe that they should perform the behaviour (*normative personal belief*), and (c) that most people in their relevant network believe they ought to conform to this behaviour as deviations from the norm could result in potential punishment (referred to as a *normative expectation*).[28] Social norms direct human action, however, norms are situationally contingent, meaning that a norm’s salience and one’s compliance to this norm are conditional upon the situation.[29] To understand and predict behaviour, it is important to know which social norms individuals find salient in particular contexts—that is, which norms are likely to be dependent on particular settings.[30, 31]

The normative system of nursing with respect to HH behaviour can be measured through learning about (a) individual’s preferences for ‘proper’ HH action, (b) expectations of others’ HH behaviour, and (c) beliefs about the expectations others have of them in this regard. We sought to identify nurses’ social norms regarding hand hygiene and whether the social norms have a causal influence on behaviour. Bicchieri (2014) devised a series of questions that diagnose, explain, and predict collective patterns of behaviour, which were adapted for the research purposes here.[28] This involves ascertaining several aspects of a normative system, including empirical expectations, normative beliefs, and normative expectations. To test empirical expectations, respondents were asked about their own beliefs regarding the prevalence of HH behaviour among their peers; respondents were asked to disclose how many nurses out of a group of ten would always practice HH at the various indications.

To test normative personal beliefs, respondents were also asked if they think they should practice HH at six various moments: (1) before entering a patient’s room, (2) when exiting a patient’s room, (3) after taking a patient’s vitals, (4) after cleaning a patient’s wound, (5) before charting in the nurse station, and (6) after talking with fellow nurses in the break room. Responses along a Likert scale from *Never* to *Always* were offered. To test normative expectations, respondents were asked if they believed that other nurses thought that they should use hand sanitizer or soap at the same moments provided above. Once again, the same Likert scale offered five response options.

#### Habit

Habits are defined as psychological tendencies to respond automatically to environmental stimuli, acquired through repeated practice in particular contexts.[32, 33] Habitual actions are triggered in response to contextual cues associated with their performance: for example, automatically putting on a seatbelt (action) after getting into the car (contextual cue) or washing hands (action) after using the toilet (contextual cue).[34] Habit strength is a continuum. Habits that are considered to be of weak or moderate strength are performed with less frequency than strong habits.[35]

Participants were asked about the strength of their HH habits using the Self-Report Habit Index (SRHI) developed by Vernplanken et al. (1994).[36] The SRHI is a tool used either as a dependent variable, or to determine or monitor habit strength without measuring behavioural frequency. It discriminates between behaviours varying in frequency and between daily vs. weekly habits. The index is based on features of habit: a history of repetition, automaticity, and expressing one’s identity. Respondents answer the degree to which they felt the statement affected them using a 5-point Likert scale (from Strongly Disagree to Strongly Agree). There is evidence that the SRHI can solicit accurate answers comparable to real behaviours.[37] The index in this case is phrased to ask respondents about practicing HH before entering and after exiting a patient’s room.

#### Motivation

Motives are evolved psychological mechanisms that help individuals choose the appropriate goal-directed behavioural strategy in response to a situation.[38] An appropriate strategy would most likely lead to a satisfactory outcome in terms of the benefits accruing from that interaction with the environment.[9] A satisfactory outcome involves an experience that is rewarding—be it a sensory pleasure, a metabolic benefit for the body, or a change to one’s place in the social world.

This research sought to identify what motivates people to practice HH. Thus, the objective of the motive questions was to determine if a person of higher status—such as a nurse manager or direct supervisor—or someone who is dependent on the nurse—such as patient—is a likely motivator of HH. The BCD’s motive mapping technique is used.[6] Motive mapping attempts to reduce psychological ‘distance’ by simulating the behavioural context using a narrative, and attempts to minimize the participant’s reflection by focusing directly on the rewards from performance.

Participants responded to three scenarios asking about how feedback is likely to influence their own HH behaviour. In each of the scenarios, participants were told that they had taken a patient’s vitals and immediately practiced HH upon exiting the room. At the end of each scenario, positive feedback regarding the practicing of HH was shared with the nurse by the nurse manger, a fellow nurse, and the patient. Respondents answered to what degree they feel this feedback makes them more likely to use hand sanitizer in the future as compared to normal usage. A five-point Likert scale measured responses.

#### Situational constraints: Vignettes

Participants were asked to judge their likely compliance to HH in varying situations known as vignettes. Vignettes are closer to real-life judgment-making situations than relatively abstract questions that are typical of most surveys. Respondents were asked to reflect on whether they would practice HH in the following situations: (1) exiting a patient’s room after taking the patient’s vitals, (2) entering a patient’s room before taking vitals, (3) exiting a patient’s room after cleaning and bandaging the patient’s diabetic foot wound, and (4) entering a patient’s room before cleaning and bandaging the patient’s foot wound. These situations were altered slightly for each follow-up question by introducing either a facilitator or a barrier to practicing HH, such as:

- Large patient load, which measures busyness
- Already wearing gloves, which measures the nurse’s inclination to practice HH when wearing protective equipment
- Being observed by the infection prevention manager, which measures higher status social influence
- Being observed by a fellow nurse, which measures peer influence
- Trying to practice hand hygiene but the dispenser is empty, which measures perception of ease
- An interruption during patient care requiring the immediate assistance of the nurse, which measures interruption
- An emergency requiring CPR, which measures reaction to emergency

Through vignettes, we sought to determine the extent to which these factors impact HH behaviour. Responses were presented on a five-point Likert scale based on the likelihoods of behavioural response.

#### Institutional factors: Safety culture and familiarity with hand hygiene

Nurse behaviour takes place within the context of hospital life. Hospitals can be considered institutions, which have an impact on the settings that occur within them. Therefore, this research sought to assess the *culture of safety* within the respondents’ institutions. It has been widely accepted that the safety culture of one’s hospital affects HHC rates.[1, 39–41] To measure the safety culture of the hospitals where the respondents work, the research team selected and modified questions from the hospital survey on patient safety culture developed by the US Agency for Healthcare Research and Quality.[42] Questions were grouped according to the safety culture dimensions they are intended to measure. Groups included: rating overall perceptions of safety, frequency of event reporting, supervisor/manager expectations and actions, teamwork within units, closeness, communication openness, feedback and communication about error, non-punitive response to error, staffing, and hospital management support. Five point Likert scales asking for agreement/disagreement and frequency were used.

Participants were also asked about their engagement and participation in past HH training and interventions, both as nursing students and as practicing professionals. In addition, participants were asked about their hospital’s own HH programs. Questions were all phrased so that a yes/no response was appropriate.

#### Modification to physical setting

Finally, the research aimed to investigate various ways to disrupt a behaviour setting, specifically by identifying how the stage and arrangement of props of the setting surrounding the act of HH serve as constraints or opportunities to practicing HH. Respondents are presented with two photos—one of a hallway in a non-descript hospital and one of a patient’s room—and then asked how both the hallway and the room could be altered to better facilitate HH. These questions allowed for open-ended responses.

### Formatting the survey

The survey was a self-administered online task. Each question was presented on its own webpage. Respondents were first asked a series of screener questions to determine if they were eligible: they had to be an acute care nurse, working in a US hospital, with a year or more of experience.

**Fig 2:**
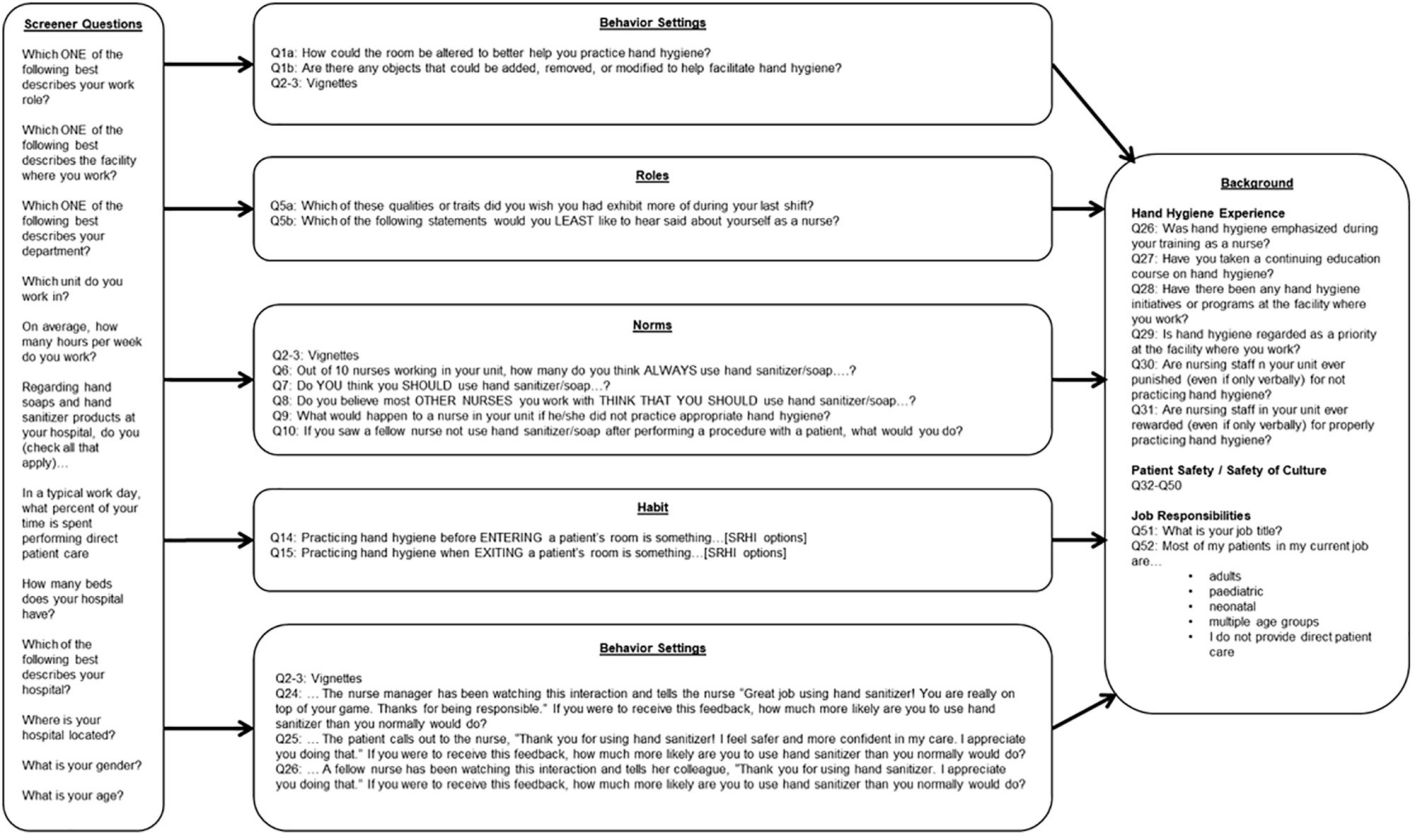
Survey flow. The survey is broken into sections; the movement of the respondent through the survey is depicted in this figure.

Those who are eligible were then presented with a series of photos related to the modification of the physical setting. These questions were asked first because the research team wanted responses that were not influenced by other questions in the survey. In addition, the photos served to ground the respondents in the survey by providing visual context. The vignettes immediately followed; the research team reasoned that the vignettes would likely solicit the most accurate responses about HH performance. As such, the vignettes were placed early in the survey so that the respondents were not biased or primed by subsequent specific queries. The professional identity questions were asked next as these questions tapped into values. Questions about norms followed and were followed by questions on habit and motivation. The final questions focused on the safety culture of the hospital as well as the respondents’ history with HH interventions and programs. A diagram of the survey questions and flow are provided in Fig 2.

### Analysis of the survey

Descriptive statistics were first used to characterize the sample. Univariate analyses were therefore first conducted to determine which variables were associated with reported levels of HHC. Next, a multivariate regression of the variable of interest—reported HH on exiting a patient room after taking vitals—was conducted on demographic, role, safety culture, and norm variables. This variable of interest was chosen as it was asked in the form of a vignette, which is closer to real-life judgment-making situations and thus provided a better sense of compliance than asking respondents directly about their HHC. In addition, this specific vignette question was used as nurses are more likely to practice HH upon exiting a room, but less likely to practice HH after conducting a low-risk procedure. As an ordinary least squares regression of outcome on predictors was inappropriate for a model with this number of predictors but only 500 observations, we performed a bidirectional stepwise procedure to build the model, using the Akaike Information Criterion (AIC) as the model-building criterion for adding or removing variables; any variable that, when removed, changed the model AIC by ≤ 1 was discarded by the procedure.

## Results

### Study population

A total of 540 surveys were completed. Table 2 summarizes selected characteristics of the participants. The median age was 49 (range: 24–70). In a typical workday, more than two-thirds of the respondents (68%) reported spending 80% or more of their time performing direct patient care. Familiarity with HH practices was indicated by 459 (85%) of respondents, who reported that HH was emphasized during professional training to be a nurse. Furthermore, the clear majority of respondents (456, or 84%) had participated in a hospital-led hand hygiene initiative before.

Summary variables were standardized before analysis. Variables included *habit*, *safety culture*, *norms*, *motivation*, *role*, *hand hygiene familiarity*, and *demographics*. Means were taken across Likert scale questions per the prescribed groupings. Sums were calculated across yes/no variables and demographic variables were encoded with a binary number system.

### Univariate analysis

The results for each of the five main potential determinants of HHC have been provided in their respective tables and figures enumerated below. Major findings have been summarized for *norms*, *habit*, and *motives*.

#### Vignettes

The results for every question in this section of the survey are included in Table 3. The most salient findings were that nurses were more likely to practice HH upon exiting a patient’s room than entering, and that when the procedure was perceived as being high-risk—such as cleaning and bandaging a wound—there was an increased likelihood of practicing HH. Most notably, 90.7% (n = 490) of nurse respondents reported being likely to practice HH upon exiting a patient’s room after cleaning and bandaging the diabetic foot wound.

**Table 3:** Responses to vignettes.

**Vitals-Vignette–Exit** You are a nurse in Normal Hospital. You need to take the vitals for Mrs. Jones in room 2. You enter the room, say hello, explain the procedure, take Mrs. Jones’ vitals, ask if she needs anything else, and then you head towards the door to leave.				
**Question**	**Response**	**N responses**	**Percent (%)**	**Corresponding figure in** S1 Fig
Base Vignette Exiting Practicing HH upon exiting the patient’s room.	Not at all likely	2	0.37	Fig A
	Slightly likely	7	1.30	
	Moderately likely	32	5.93	
	Very likely	124	22.96	
	Extremely likely	375	69.44	
Busyness Practicing HH when leaving the patient’s room with other demanding tasks on the mind	Much less likely	11	2.04	Fig B
	Somewhat less likely	71	13.15	
	No difference	357	66.11	
	Somewhat more likely	33	6.11	
	Much more likely	68	12.59	
Gloves Practicing HH after taking off gloves	Much less likely	8	1.48	Fig B
	Somewhat less likely	53	9.81	
	No difference	354	65.56	
	Somewhat more likely	44	8.15	
	Much more likely	81	15.00	
Peer Influence Practicing HH when seeing a fellow nurse outside the patient’s room	Much less likely	0	0	Fig B
	Somewhat less likely	11	2.04	
	No difference	365	67.59	
	Somewhat more likely	86	15.93	
	Much more likely	78	14.44	
Higher Status Social Influence Practicing HH when seeing the hospital’s Infection Prevention Director outside the patient’s room	Much less likely	0	0	Fig B
	Somewhat less likely	0	0	
	No difference	257	47.59	
	Somewhat more likely	70	12.96	
	Much more likely	213	39.44	
Higher Status Modelling Practicing HH when leaving the patient’s room even though the Nurse Manager did not practice HH	Much less likely	2	0.37	Fig B
	Somewhat less likely	9	1.67	
	No difference	351	65.00	
	Somewhat more likely	69	12.78	
	Much more likely	109	20.19	
Empty Dispenser Practicing HH when there is an empty ABHR dispenser	Much less likely	38	7.04	Fig B
	Somewhat less likely	162	30.00	
	No difference	248	45.93	
	Somewhat more likely	37	6.85	
	Much more likely	55	10.19	
Interruption Practicing HH when interrupted upon leaving a patient’s room	Much less likely	30	5.56	Fig B
	Somewhat less likely	117	21.67	
	No difference	296	54.81	
	Somewhat more likely	35	6.48	
	Much more likely	62	11.48	
Emergency Practicing HH when exiting the patient’s room to attend to an emergency	Much less likely	118	21.85	Fig B
	Somewhat less likely	162	30.00	
	No difference	188	34.81	
	Somewhat more likely	30	5.56	
	Much more likely	42	7.78	
**Vitals-Vignette–Entry** Now instead of exiting Mrs. Jones’s room, you are entering her room to take her vitals.				
**Question**	**Response**	**N responses**	**Percent (%)**	**Corresponding figure**
Base Vignette Entry Practicing HH before entering patient’s room	Not at all likely	6.	1.11	Fig A
	Slightly likely	30.	5.56	
	Moderately likely	64.	11.85	
	Very likely	132.	24.44	
	Extremely likely	308.	57.04	
Patient’s request Practicing HH upon patient’s request	Much less likely	1	0.19	Fig C
	Somewhat less likely	0	0	
	No difference	230	42.59	
	Somewhat more likely	37	6.85	
	Much more likely	272	50.37	
Empty Dispenser Practicing HH when there is an empty ABHR dispenser	Much less likely	37	6.85	Fig C
	Somewhat less likely	145	26.85	
	No difference	270	50.00	
	Somewhat more likely	34	6.30	
	Much more likely	54	10.00	
Gloves Practicing HH before putting on gloves	Much less likely	47	8.70	Fig C
	Somewhat less likely	134	24.81	
	No difference	285	52.78	
	Somewhat more likely	27	5.00	
	Much more likely	47	8.70	
**Cleaning Wound- Vignette–Exit** You are a nurse at Normal Hospital. You are cleaning and bandaging Mr. Robinson’s diabetic foot. After finishing the procedure, you take off your gloves, and then say goodbye to Mr. Robinson.				
**Question**	**Response**	**N responses**	**Percent (%)**	**Corresponding figure**
Base Vignette Exit How likely are you to practice hand hygiene upon exiting the room?	Not at all likely	0	0	Fig A
	Slightly likely	4	0.74	
	Moderately likely	4	0.74	
	Very likely	42	7.78	
	Extremely likely	490	90.74	
Busyness Practicing HH when leaving the patient’s room with other demanding tasks on the mind	Much less likely	1	0.19	Fig D
	Somewhat less likely	11	2.04	
	No difference	382	70.74	
	Somewhat more likely	36	6.67	
	Much more likely	110	20.37	
Peer Influence Practicing HH when seeing a fellow nurse outside the patient’s room	Much less likely	0	0.	Fig D
	Somewhat less likely	3	0.56	
	No difference	389	72.04	
	Somewhat more likely	53	9.81	
	Much more likely	95	17.59	
Higher Status Social Influence Practicing HH when seeing the hospital’s Infection Prevention Director outside the patient’s room	Much less likely	0	0	Fig D
	Somewhat less likely	1	0.19	
	No difference	316	58.52	
	Somewhat more likely	51	9.44	
	Much more likely	172	31.85	
Higher Status Modelling Practicing HH when leaving the patient’s room even though the Nurse Manager did not practice HH	Much less likely	1	0.19	Fig D
	Somewhat less likely	5	0.93	
	No difference	384	71.11	
	Somewhat more likely	44	8.15	
	Much more likely	106	19.63	
Empty Dispenser Practicing HH when there is an empty ABHR dispenser	Much less likely	6	1.11	Fig D
	Somewhat less likely	70	12.96	
	No difference	347	64.26	
	Somewhat more likely	32	5.93	
	Much more likely	85	15.74	
Interruption Practicing HH when interrupted upon leaving a patient’s room	Much less likely	4	0.74	Fig D
	Somewhat less likely	75	13.89	
	No difference	351	65.00	
	Somewhat more likely	34	6.30	
	Much more likely	76	14.07	
Emergency Practicing HH when exiting the patient’s room to attend to an emergency	Much less likely	57	10.56	Fig D
	Somewhat less likely	125	23.15	
	No difference	260	48.15	
	Somewhat more likely	32	5.93	
	Much more likely	66	12.22	
**Cleaning Wound- Vignette–Enter** Now instead of *exiting* Mr. Robinson’s room, you are entering his room to clean and reapply his bandages. After reading each scenario, please answer the following questions.				
**Question**	**Response**	**N responses**	**Percent (%)**	**Corresponding figure**
Base Vignette Entry Practicing HH before entering patient’s room	Not at all likely	4	0.74	Fig A
	Slightly likely	18	3.33	
	Moderately likely	48	8.89	
	Very likely	116	21.48	
	Extremely likely	354	65.56	
Patient’s request Practicing HH upon patient’s request	Much less likely	4	0.74	Fig E
	Somewhat less likely	18	3.33	
	No difference	48	8.89	
	Somewhat more likely	116	21.48	
	Much more likely	354	65.56	
Empty Dispenser Practicing HH when there is an empty ABHR dispenser	Much less likely	3	0.56	Fig E
	Somewhat less likely	2	0.37	
	No difference	264	48.89	
	Somewhat more likely	45	8.33	
	Much more likely	226	41.85	
Gloves Practicing HH before putting on gloves	Much less likely	18	3.33	Fig E
	Somewhat less likely	110	20.37	
	No difference	299	55.37	
	Somewhat more likely	40	7.41	
	Much more likely	73	13.52	

#### Norms

The results for empirical expectations, normative personal beliefs, and normative expectations have been presented in Table 4. Regarding *empirical expectations*, respondents felt that most nurses practiced hand hygiene before entering a patient’s room, when exiting a patient’s room, after taking a patient’s vitals, and after cleaning a patient’s wound. Concerning *normative personal beliefs*, for each moment apart from charting, most respondents claimed that HH should always be practiced. Of the 540 respondents, 81.7% (n = 441) of respondents said it should always be practiced before entering a patient’s room, 90.4% (n = 488) when exiting a patient’s room, 75.6% (n = 408) after taking patient’s vitals, and 98.7% (n = 533) after cleaning a patient’s wound. With *normative expectations*, over 50% of respondents claimed that most other nurses always think that one should practice hand hygiene before entering a patient’s room, when exiting a patient’s room, after taking a patient’s vitals, and after cleaning a patient’s wound. [Figs H and I in the S1 Fig display the results.]

**Table 4:** Responses to norm questions.

**Empirical Expectations** Number of nurses out of 10 that always practice hand hygiene:				
**Questions**	**Response**	**N response**	**Percent (%)**	**Corresponding Figure in** S1 Fig
before entering a patient’s room?	0	7	1.29	Fig H
	1	9	1.67	
	2	27	5.00	
	3	23	4.26	
	4	14	2.59	
	5	91	16.85	
	6	32	5.93	
	7	52	9.63	
	8	128	23.70	
	9	82	15.19	
	10	75	13.89	
when exiting a patient’s room?	0	4	0.74	Fig H
	1	1	0.19	
	2	10	1.85	
	3	6	1.11	
	4	10	1.85	
	5	45	8.33	
	6	36	6.67	
	7	52	9.63	
	8	146	27.04	
	9	116	21.48	
	10	114	21.11	
after taking a patient’s vitals?	0	14	2.59	Fig H
	1	11	2.037	
	2	37	6.85	
	3	18	3.33	
	4	23	4.26	
	5	101	18.70	
	6	43	7.96	
	7	46	8.52	
	8	103	19.07	
	9	65	12.04	
	10	79	14.63	
after cleaning a patient’s wound?	0	2	0.37	Fig H
	1	2	0.37	
	2	2	0.37	
	3	2	0.37	
	4	0	0.	
	5	10	1.85	
	6	4	0.74	
	7	9	1.67	
	8	39	7.22	
	9	96	17.78	
	10	374	69.26	
before charting in the nurse station?	0	53	9.82	Fig H
	1	22	4.07	
	2	48	8.89	
	3	17	3.15	
	4	31	5.74	
	5	108	20.00	
	6	39	7.22	
	7	47	8.70	
	8	77	14.26	
	9	45	8.33	
	10	53	9.82	
after talking to a colleague in the hallway?	0	156	28.89	Fig H
	1	40	7.41	
	2	67	12.41	
	3	31	5.74	
	4	25	4.63	
	5	89	16.48	
	6	22	4.07	
	7	24	4.44	
	8	38	7.04	
	9	19	3.52	
	10	29	5.37	
**Normative Personal Beliefs** Do you think you should practice hand hygiene:				
**Questions**	**Response**	**N response**	**Percent (%)**	**Corresponding Figure**
before entering a patient’s room?	Never	0	0	Fig I
	Seldom	11	2.04	
	About half the time	12	2.22	
	Usually	76	14.07	
	Always	441	81.67	
when exiting a patient’s room?	Never	0	0	Fig I
	Seldom	2	0.37	
	About half the time	8	1.48	
	Usually	42	7.78	
	Always	488	90.37	
after taking a patient’s vitals?	Never	3	0.56	Fig I
	Seldom	13	2.41	
	About half the time	33	6.11	
	Usually	83	15.37	
	Always	408	75.56	
after cleaning a patient’s wound?	Never	0	0	Fig I
	Seldom	0	0	
	About half the time	2	0.37	
	Usually	5	0.93	
	Always	533	98.70	
before charting in the nurse station?	Never	23	4.26	Fig I
	Seldom	57	10.56	
	About half the time	71	13.15	
	Usually	150	27.78	
	Always	239	44.26	
**Normative Expectations** Do you believe that most other nurses think that you should practice hand hygiene:				
**Questions**	**Response**	**N response**	**Percent (%)**	**Corresponding Figure**
before entering a patient’s room?	Never	2	0.37	Fig J
	Seldom	13	2.407	
	About half the time	51	9.444	
	Usually	136	25.185	
	Always	338	62.593	
when exiting a patient’s room?	Never	1	0.185	Fig J
	Seldom	1	0.185	
	About half the time	32	5.926	
	Usually	101	18.704	
	Always	405	75.	
after taking a patient’s vitals?	Never	9	1.667	Fig J
	Seldom	30	5.556	
	About half the time	78	14.444	
	Usually	148	27.407	
	Always	275	50.926	
after cleaning a patient’s wound?	Never	0	0.	Fig J
	Seldom	1	0.185	
	About half the time	8	1.481	
	Usually	43	7.963	
	Always	488	90.37	
before charting in the nurse station?	Never	37	6.852	Fig J
	Seldom	92	17.037	
	About half the time	126	23.333	
	Usually	140	25.926	
	Always	145	26.852	
after talking with fellow nurses in the break room?	Never	82	15.185	Fig J
	Seldom	146	27.037	
	About half the time	116	21.481	
	Usually	86	15.926	
	Always	110	20.37	

#### Habit

**Fig 3:**
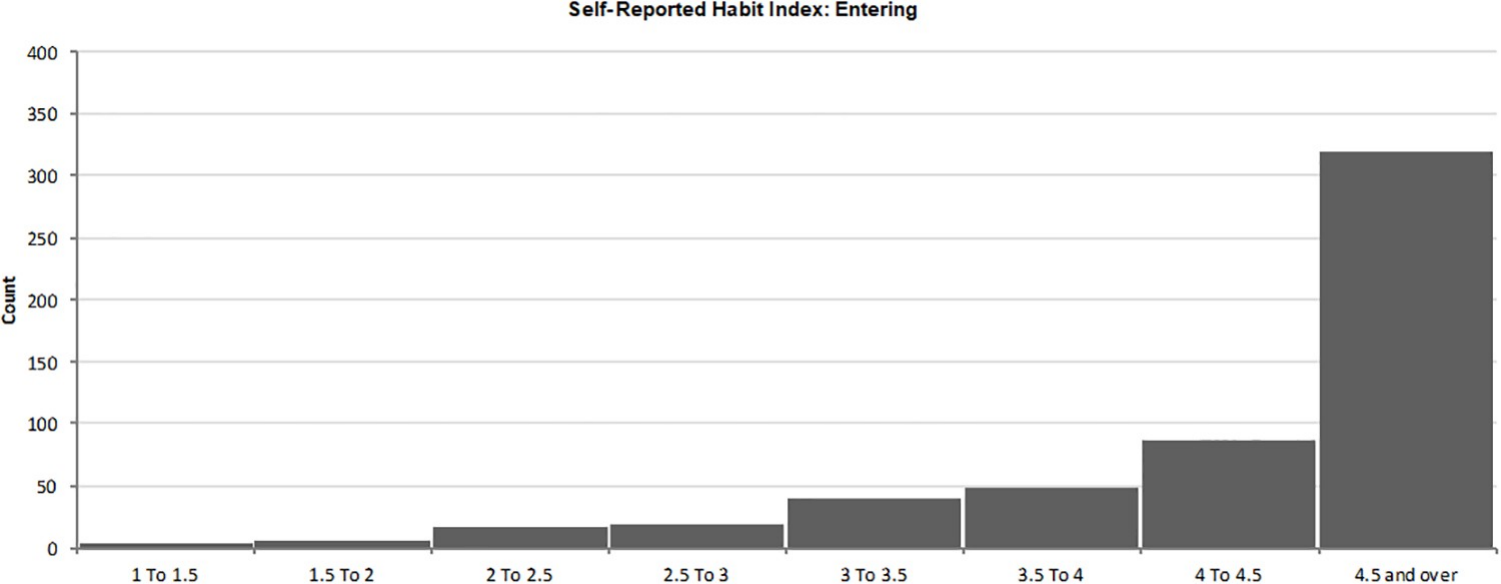
Self-Reported habit index: Entering.

**Fig 4:**
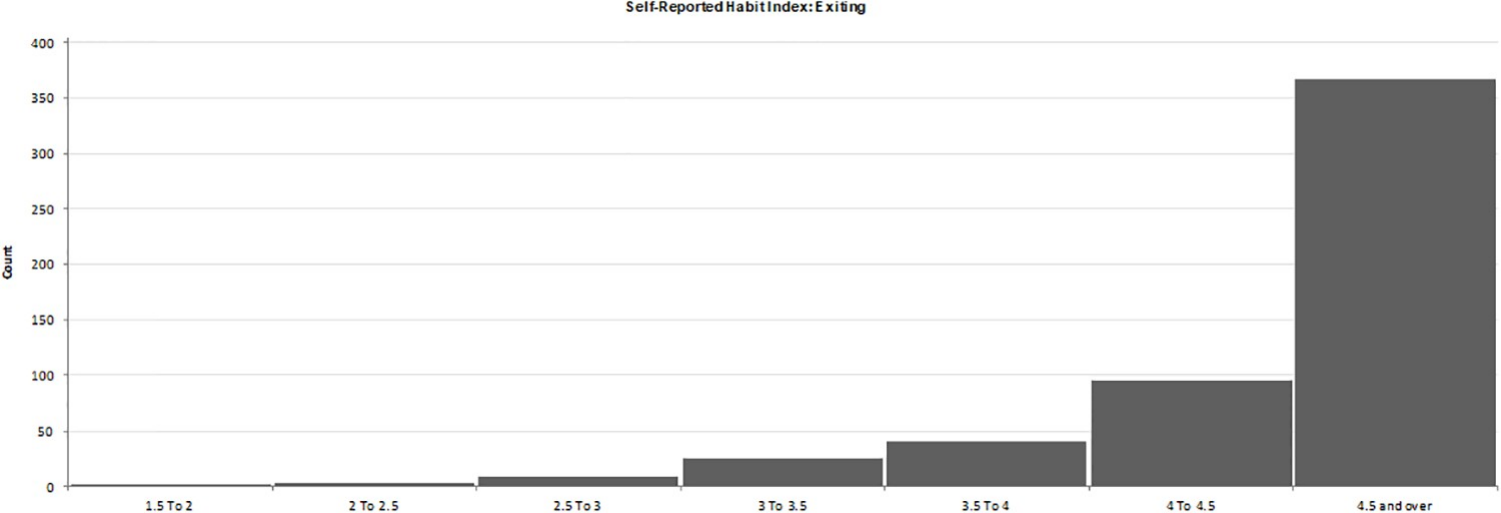
Self-Reported habit index: Exiting.

Respondents answered the SRHI about practicing HH before entering a patient’s room and after exiting a patient’s room. Responses were made on five point Likert scales anchored by the terms strongly agree-strongly disagree and were coded such that high values indicated strong habits (1 = strongly disagreeing and 5 = strongly agreeing). The means of the questions were calculated, and these in turn became the habit strength scores. Regarding HH upon entering a room, 59.1% (n = 319) of respondents had a score of 4.5 or over (Fig 3). In the case of exiting, 68.0% (n = 367) of respondents had a habit strength score of 4.5 and over (Fig 4).

#### Motives

Upon receiving feedback from nurse managers and fellow nurses, 50.7% (n = 274) of participants and 55.4% (n = 299) said that there would be no difference in future HH action, respectively. Regarding receiving feedback from patients, 59.3% (n = 320) respondents said that feedback would positively impact their HH behaviour in the future. Results are summarized in Table 5.

**Table 5:** Responses to motives questions.

MOTIVATION				
Questions	Response	N response	Percent (%)	Corresponding Figures [S1 Fig]
Feedback from nurse manager	Much Less Likely	2	0.37	Fig K
	Somewhat Less Likely	1	0.19	
	No Difference	274	50.74	
	Somewhat More Likely	114	21.11	
	Much More Likely	149	27.59	
Feedback from patient	Much Less Likely	1	0.19	Fig K
	Somewhat Less Likely	0	0.	
	No Difference	299	40.56	
	Somewhat More Likely	111	22.59	
	Much More Likely	129	36.67	
Feedback from colleague	Much Less Likely	1	0.19	Fig K
	Somewhat Less Likely	0	0.	
	No Difference	299	55.37	
	Somewhat More Likely	111	20.56	
	Much More Likely	129	23.89	

#### Safety culture

The results for each question in this section of the survey are included in Table 6.

**Table 6:** Responses to questions about safety culture.

**Rating Overall Perceptions of Safety**			
**Questions**	**Response**	**N response**	**Percent (%)**
Patient safety is never sacrificed to get more work done.	Strongly disagree	33	6.00
	Disagree	131	24.2
	Neither agree nor disagree	96	17.8
	Agree	168	31.1
	Strongly Agree	112	20.7
Our procedures and systems are good at preventing errors from happening.	Strongly disagree	10	1.9
	Disagree	50	9.3
	Neither agree nor disagree	73	14.6
	Agree	285	52.8
	Strongly Agree	122	22.6
When a mistake is made that could harm the patient, but does not, how often is this reported?	Always	89	16.5
	Usually	234	43.3
	Half the time	155	28.7
	Seldom	59	10.9
	Never	3	0.56
**Supervisor and Manager Expectations and Action**			
**Questions**	**Response**	**N response**	**Percent (%)**
My supervisor/manager overlooks patient safety problems that repeatedly happen.	Strongly disagree	100	18.5
	Disagree	194	35.9
	Neither agree nor disagree	68	12.4
	Agree	111	20.6
	Strongly Agree	67	12.4
My supervisor/manager seriously considers staff suggestions for improving patient safety.	Strongly disagree	16	2.96
	Disagree	56	10.4
	Neither agree nor disagree	99	18.3
	Agree	252	46.7
	Strongly Agree	117	21.7
My supervisor/manager says a good word when observing a job done according to established patient safety procedures.	Strongly disagree	25	4.6
	Disagree	67	12.4
	Neither agree nor disagree	129	23.9
	Agree	219	40.6
	Strongly Agree	100	18.5
**Teamwork Within Units**			
**Questions**	**Response**	**N response**	**Percent (%)**
Nurses in our unit help each other out regularly.	Strongly disagree	6	1.1
	Disagree	15	2.8
	Neither agree nor disagree	22	4.1
	Agree	244	45.2
	Strongly Agree	253	46.9
I can depend on getting help from other nurses.	Strongly disagree	5	0.92
	Disagree	25	4.6
	Neither agree nor disagree	38	7.0
	Agree	254	47.0
	Strongly Agree	218	40.5
In this unit, people treat each other with respect.	Strongly disagree	8	1.5
	Disagree	24	4.4
	Neither agree nor disagree	46	8.5
	Agree	293	54.3
	Strongly Agree	169	31.3
**Closeness**			
**Questions**	**Response**	**N response**	**Percent (%)**
Some of my closest friends are my work colleagues.	Strongly disagree	18	3.3
	Disagree	66	12.2
	Neither agree nor disagree	111	20.6
	Agree	207	38.3
	Strongly Agree	138	25.6
**Communication Openness**			
**Questions**	**Response**	**N response**	**Percent (%)**
Staff will freely speak up if they see something that may negatively affect patient care.	Always	117	21.7
	Usually	284	52.6
	Half the time	107	19.8
	Seldom	28	5.2
	Never	4	0.7
Staff feel free to question the decisions or actions of those with more authority.	Strongly disagree	20	3.7
	Disagree	103	19.1
	Neither agree nor disagree	134	24.8
	Agree	202	37.4
	Strongly Agree	81	15.0
Staff are afraid to ask questions when something does not seem right.	Strongly disagree	48	8.9
	Disagree	241	44.6
	Neither agree nor disagree	134	24.8
	Agree	84	15.6
	Strongly Agree	33	6.1
**Feedback and Communication About Error**			
**Questions**	**Response**	**N response**	**Percent (%)**
In this unit, we discuss ways to prevent errors from happening again.	Always	117	21.7
	Usually	284	52.6
	Half the time	107	19.8
	Seldom	28	5.2
	Never	4	0.74
**Staffing**			
**Questions**	**Response**	**N response**	**Percent (%)**
We sometimes work in “crisis mode” trying to do too much, too quickly.	Strongly disagree	5	0.93
	Disagree	48	8.9
	Neither agree nor disagree	67	12.4
	Agree	289	53.5
	Strongly Agree	131	24.3
Hospital management seems interested in patient safety only after an adverse event happens	Strongly disagree	40	7.4
	Disagree	136	25.2
	Neither agree nor disagree	110	20.4
	Agree	164	30.4
	Strongly Agree	90	16.7

### Multivariate regression

Presented in Table 7 are the results from the bidirectional stepwise procedure to analyse the relationships between various predictors and the outcome: reported HH on exiting a patient room after taking vitals. Included in the table are only the variables which met the selection criteria. Values are provided for the regression Estimate, as well as its Standard Error, T-value, and Pr(>|t|) coefficients. Coefficients were assigned to each predictor; the sign on the coefficient (positive or negative) provides the direction of the effect of the predictor on the outcome variable.

**Table 7:** Stepwise regression model results.

	Estimate	Standard Error	T value	Pr(>|t|)
**INTERCEPT**	3.228	0.511	6.315	5.84E-10
**HOSPITAL LEVEL FACTORS**				
Openness of communication	0.117	0.049	2.388	0.017
**UNIT LEVEL FACTORS**				
Type of Unit: Emergency Department	-0.213	0.086	-2.496	0.013
Hours worked per week	-0.013	0.005	2.467	0.014
Percent of time for patient care	0.102	0.040	2.520	0.012
Percent of time spent interacting with patient	0.004	0.002	2.366	0.018
Percent of time spent on professional interactions	0.019	0.005	3.747	0.0002
**INDIVIDUAL LEVEL FACTORS**				
Which quality did you wish you had exhibited more during your last shift?				
Good communication skills	-0.120	0.061	-1.975	0.049
Stress management	0.135	0.058	2.334	0.020
Which quality would you least like to hear during your last shift?				
Unsure of self as nurse	-0.128	0.060	-2.138	0.033
**NORMS**				
Out of 10 nurses working in your unit, how many do you think always use hand sanitizer or soap…				
after talking to colleague in hallway	0.041	0.010	1.970	0.049
after cleaning a patient’s wound	-0.071	0.024	-2.935	0.003
after taking patient’s vitals	0.041	0.014	2.823	0.005
when exiting a patient’s room	0.073	0.020	3.684	0.0003

## Discussion

### Univariate analysis

#### Vignettes

The reported higher likelihood of practicing HH upon performing a high-risk procedure as compared to a low-risk procedure aligns with the literature which shows that HHC is greater when involving higher-risk tasks.[2, 43, 44] In addition, nurses reported being more likely to practice HH upon exiting a patient’s room than entering, which is interpreted as nurses practicing HH as a form of self-protection.[44]

#### Role

Nurses work in close relationships with patients who are vulnerable and largely dependent on the nurse for care.[45] Nurses work with one another and on inter-professional healthcare teams to deliver care and provide support. Fagermoen’s (1997) proposed theoretical model for professional identity of nurses maintains that nurses’ perceptions of the ‘professional self’ focuses on both *other-oriented* and *self-oriented values*.[45] *Other-oriented values* encompass the nurse’s actions on behalf of the patient’s well-being and the interactions with patients in providing care. *Self-oriented work values* include work performance and collaboration with other professionals. While *self-oriented work values* directly impact the self, these values also affect the care delivered. For instance, better stress management can lead to a nurse feeling more confident, capable, and in control, which can then lead to better care delivered.

When asked which values the participants wish they had exhibited more of during their last shift, the traits most widely selected were those of *self-oriented values* such as stress management, patience, good communication, and physical and mental endurance. These in turn impact *other-oriented values* to a degree since work performance directly influences the kind of care delivered. *Other-oriented values* are the foundation of nursing care and an integral part of the nurses’ relationships with patients. Areas of improvement could be seen in how nurses engage in the work-setting and the actualization of the *other-oriented values*. When asked what the nurses would least like to hear said about them, the top responses were about the inadequacy in the delivery of care. This again demonstrates how integral *other-oriented values* are to the discipline of nursing.

#### Norms

There is agreement amongst participants as to when to practice HH—upon entering and exiting a patients’ room and after performing a procedure such as vitals or cleaning a wound. It is apparent that participants believed these to be norms, and believed others to hold the same norms in addition to conforming to such norms. This suggests that HH indications are well understood and agreed upon by nurses.

#### Habit

Habit is the cognitive mechanism by which actions occur reflexively and in a fixed sequence.[46] Habit scores were quite high, which is not unexpected for a behaviour that is practiced many times a day. This suggests that the SRHI may not be useful in measuring behaviour that is already being practiced intensively.

#### Motives

Over half of participants indicated that receiving feedback from a patient or a colleague would likely lead to an increase in future HH action. There is evidence that HH behaviour of HCWs is positively influenced by the presence and proximity of peers.[47, 48] Regarding patients, patient involvement in supporting their own safety has been widely discussed. [49–51]. Patient involvement in HH—such as praising HCWs for practicing HH or reminding HCWs to wash their hands—and its impact on HH behaviour has not been extensively studied [51], but our results show that it would be acceptable to HCWs for patients to recognize nurses for practicing HH.

### Multivariate regression

The variable of interest was the reported HHC upon exiting a patient’s room after taking their vitals. This question had the most variance in responses. The regression analysis shows that reported HHC is a function of specific variables at all possible levels: the hospital, unit, and individual. At the hospital level, increased *openness of communication*—which was asked about in the safety culture portion of the survey—led to a higher reporting of HHC. There is evidence that features of a hospital’s safety climate are related to how well standard precautions and safety practices, such as HH, are adhered to.[52–54] *Communication openness* is a component of a hospital’s patient safety culture and is defined as the extent to which the staff freely speak up if they see something that may negatively affect a patient and/or question those with more authority.[40, 55] A core tenet behind *communication openness* is that all have a responsibility to speak out when certain actions, objects, or processes pose danger to the safety of the patient and others, and those who speak out should be able to do so without fear of being reprimanded. It could be surmised that those who are comfortable enough to speak out about threats to patient safety would also act on their own accord to protect patient safety by practicing HH at the proper indications.

At the unit level, the type of hospital unit played a role in the HHC reported—overall, participants who work in an emergency department reported lower HHC rates. This could be attributed to the fact that nurses must respond to various unpredictable situations that could be life-threatening to the patient, and the patient’s need for immediate attention and care is put first before practicing HH. Practicing HH in an emergency could be perceived as dilatory. This could also be because the emergency department is an environment with a high density of invasive procedures that require glove usage, and there is evidence that glove usage is inversely correlated with adequate HH. [1, 56, 57]

An interesting finding was that nurses who indicated having a higher proportion of shift time allocated to interaction with patients and with fellow healthcare professionals reported higher HHC. More time spent with a patient could lead to more opportunities to practice HH and thus more events completed. However, this challenges the notion that the higher the demand for hygiene (the more opportunities to practice it), the lower the adherence rates. Nevertheless, the more time spent with other HCWs could result in a nurse feeling the ‘watching eyes’ effect thus leading to increased HHC. More time with the patient could also result in the nurse bonding with the patient and is thus more cognisant of practicing HH to ensure the patient’s safety.

At the individual level, one’s personal ability to manage subjectively important aspects of the professional role—such as stress management, communication skills, and being confident in one’s self as a nurse—leads to increased reporting of HHC. All the individual-level variables in the analysis could be defined as *other-oriented* to a degree as presumably successful stress management can lead to providing better care. The significant individual variables show *other-oriented* values involving care and communication as being of highest professional importance to nurses, and this orientation fosters better HH.

It has been noted in the literature that poor working conditions, increased levels of stress, and insufficient communication have a direct negative impact on the quality of nursing and have severe consequences for patients.[58–61] In addition, low HHC can result from fatigue or burnout. As a nurses’ shift progresses, HHC declines towards the end of the shift.[62] Continuous long shifts can lead to nurse burnout which in turn has been associated with increased HAI levels.[63] Thus, nurses who feel in control, confident in their abilities, supported, and have lower stress levels can better focus on and execute safety procedures such as HH.

### Limitations

Surveys administered to HCWs are relatively inexpensive and allow for HCWs to focus and reflect on their own practices. However, self-report of infection prevention can be flawed, especially as reported HH practices and actual HH practice can differ significantly.[54, 64, 65] In using vignettes, we may have reduced socially desirable responses by allowing participants to report their HH practice and the practices of others through the vignette character(s) and situations.[65, 66] This may have reduced the potential for disparity between reported and actual behaviour. Additionally, generalizability of the findings may be limited by certain characteristics of the sample, achieved through online data recruitment. This limitation was addressed by administering the survey online, which allowed for us to collect responses from a wide variety of participants located in different regions and hospitals of the United States with varying degrees of experience and specialisation.

## Conclusion

Formative research was undertaken to assess the potential impact of several unexamined factors that could influence HH among nurses: *professional role and status*, *social affiliation*, *social norms*, and *physical modifications to the work environment*, as well as *institutional factors* (like safety climate). A survey questionnaire looked at how these factors influence nurses’ reported HHC and also sought to identify barriers and levers to HH. Multivariate regression modelling suggested that HHC was most likely to be a function of a hospital management’s ‘openness’, perceived performance by peers, increased interactions with patients and other staff members, and the reduction in stress, busyness, and cognitive load associated with role performance. Thus, a powerful and effective intervention focusing on nurses’ HHC should address improving communication openness, consider the impact of perceived performance by peers, increase interactions with patients and staff, and determine how to reduce the stress and cognitive load associated with role performance. Use of Behaviour Centred Design increased the informativeness of the survey tool, and could be used more widely in formative research studies.

## Supporting information

S1 File Search strings. Concepts and their corresponding search strings.
(DOCX)

S2 File Survey tool. (DOCX)

S1 Fig Results presented in figures. (DOCX)

## Abbreviations

ABHR: alcohol-based hand rub
BCD: Behaviour Centred Design
BCTs: behaviour change techniques
HAIs: healthcare associated infections
HCWs: healthcare workers
HH: hand hygiene
HHC: hand hygiene compliance
ICU: intensive care unit
SRHI: Self-reported habit index
