# Supplementary material for: Determinants of hand hygiene compliance among nurses in US hospitals: A formative research study
Source: PLoS One. 2020 Apr 7;15(4):e0230573. doi: 10.1371/journal.pone.0230573 (PMC7138309; doi:10.1371/journal.pone.0230573)
Supplement: S1 File — Concepts and their corresponding search strings. (DOCX) [file pone.0230573.s001.docx]

| **Concept** | **Search Strings** |
| --- | --- |
| Behaviour Change | 1. behavio#r change OR behavio#r change ADJ3 theor* OR behavio#r change ADJ3 principle* OR behavio#r change ADJ3 model* OR behavio#r change ADJ3 strateg* OR behavio#r change ADJ3 attitude* OR behavio#r change ADJ3 value* OR behavio#r change ADJ3 health* OR behavio#r change ADJ3 promotion* OR behavio#r change ADJ3 maint* OR behavio#r change ADJ3 understand* OR social marketing OR health promotion 2. Subject Headings/ Index for ‘behavior change’ (Medline: health promotion/ OR health behaviour/ OR health education/ OR health knowledge, attitudes, practice/ OR marketing of health services/ OR social marketing/) 3. Search 1 OR Search 2 |
| Hand Hygiene Compliance (HHC) | 1. hand hygiene compl* OR hand hygiene adhere* OR hand hygiene ADJ3 compl* OR hand hygiene ADJ3 adhere* 2. Subject Headings/Index for ‘hand hygiene compliance’ (Medline: hand hygiene/ OR hand disinfection/) 3. Search 2 OR Search 2 |
| Healthcare Workers (HCWs) | 1. nurse* or nursing or physician* or doctor* 2. Subject Headings/Index for ‘health care personnel’ (Medline: health personnel/ OR faculty, medical/ OR faculty, nursing/ OR infection control practioners/ OR medical staff/ OR nurses/ OR nursing staff/ OR physicians/) 3. Search 1 OR Search 2 |
| Initiatives | 1. intervention* OR program* OR activit* OR technique* OR technolog* OR protocol* OR initiative* OR campaign* |
| Hand Hygiene | 1. handwashing OR hand wash OR handwash OR hand hygiene 2. Subject Headings/Index for ‘hand hygiene’ (Medline: hand hygiene/ OR hand disinfection/) |
| Healthcare-Associated Infection | 1. Healthcare associated infection* OR health care associated infection* OR healthcare-associated infection* OR health care-associated infection* OR healthcare acquired infection* OR health care acquired infection* OR healthcare-acquired infection* OR health care-acquired infection* 2. central line-associated bloodstream infections* OR CLABSI* OR catheter- associated urinary tract infection* OR CAUTI* OR surgical site infection* OR SSI*OR methicillin- resistant Staphylococcus aureus OR MRSA OR Clostridium difficile infection* OR C. difficile infection* 3. Nosocomial pathogen* 4. Search 1 OR Search 2 OR 3 |
