# Supplementary material for: Determinants of hand hygiene compliance among nurses in US hospitals: A formative research study
Source: PLoS One. 2020 Apr 7;15(4):e0230573. doi: 10.1371/journal.pone.0230573 (PMC7138309; doi:10.1371/journal.pone.0230573)
Supplement: S2 File — (DOCX) [file pone.0230573.s002.docx]

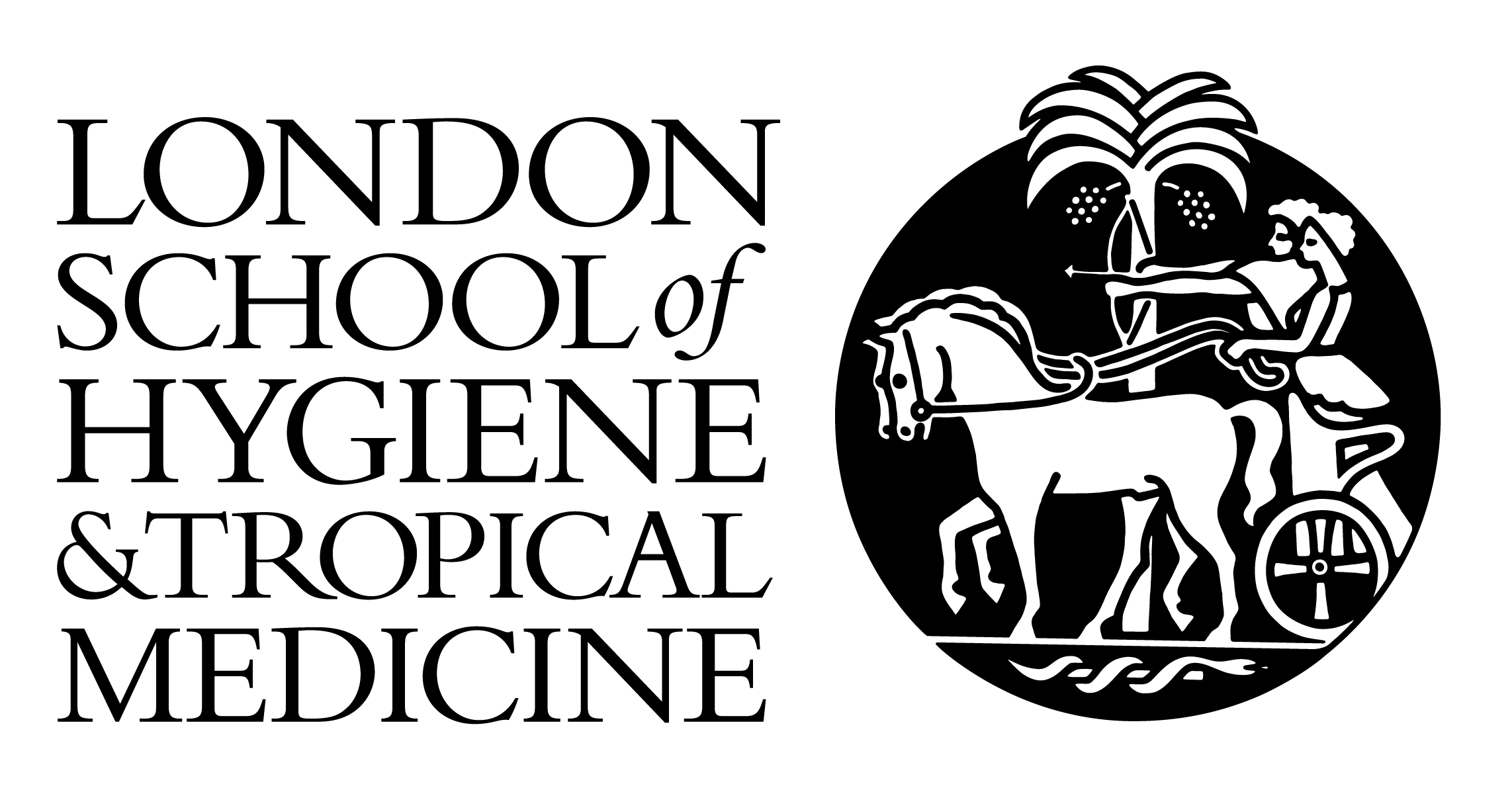
GOJO acute care Nurse HH survey (002-237), 11/2015

**CONFIDENTIAL**

**NOT TO BE DISTRIBUTED**

GOJO Contact: Jeff Quinn, 913-284-1793 CONFIDENTIAL

**SECTION A**

**Imagine you are a nurse in Community General Hospital. You enter the patient room to draw the patient’s blood for morning labs. *Please look at the photo of the hallway/entrance to the patient room and the photo of the patient room and answer the following questions.***

1. **How could the room be altered to better help you practice hand hygiene? [open-ended response]**
2. **Are there any objects that could be added, removed, or modified to help facilitate hand hygiene? [open-ended response]**

**Hallway and entrance to the patient room.**

**SECTION B**

**Now you will be asked to imagine yourself in a particular situation during a regular day at work. *Read the scenario and answer the following questions.***

**
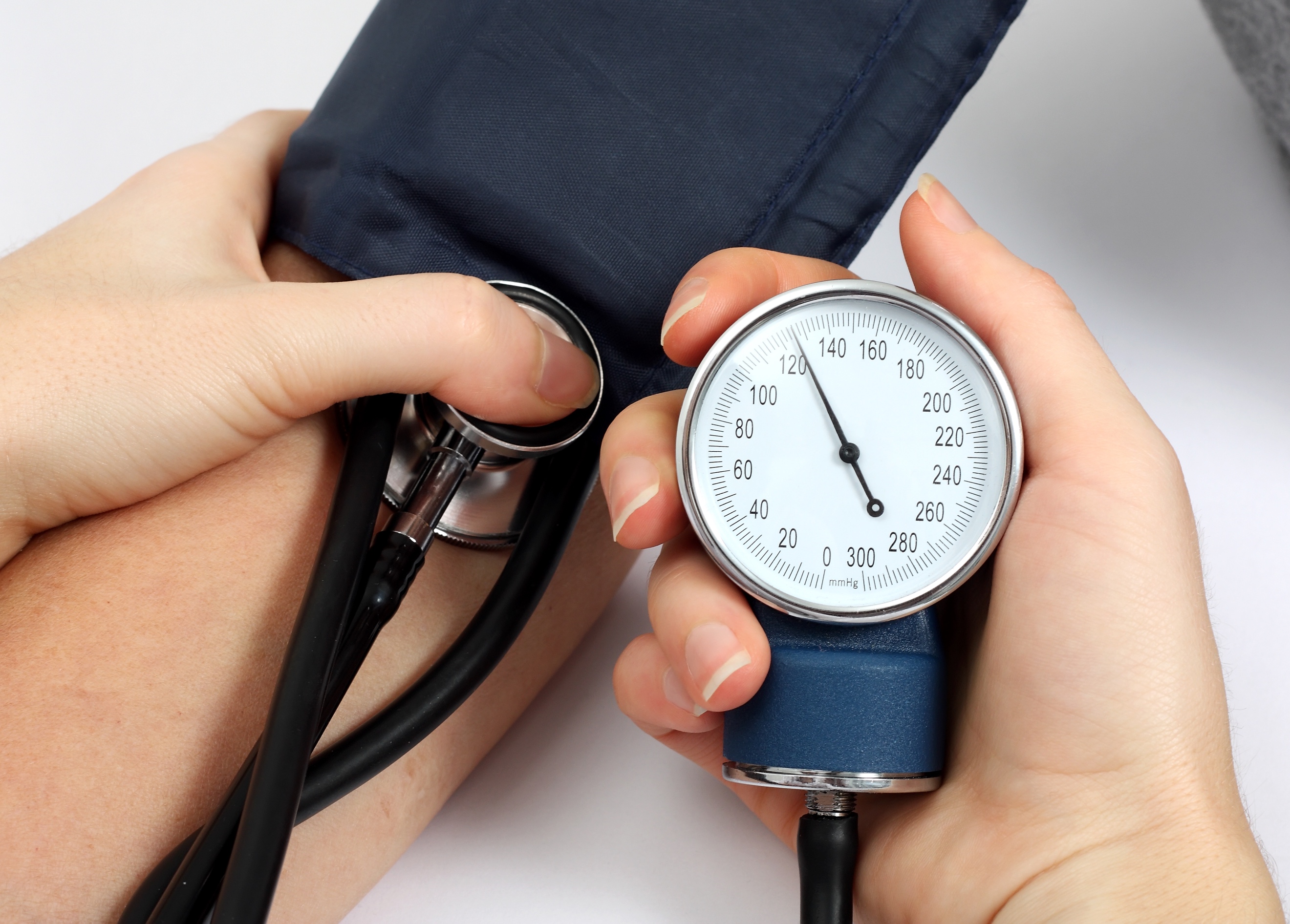
**

1. You are a nurse in Normal Hospital. You need to take the vitals for Mrs. Jones in room 2. You enter the room, say hello, explain the procedure, take Mrs. Jones’ vitals, ask if she needs anything else, and then you head towards the door to leave.


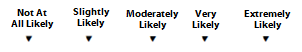


**How likely are you to practice hand hygiene upon exiting the room?**

1. The unit is short-staffed today and you are busier than normal. You have to attend to two other patients, you need to debrief the attending physician, and fill out your shift report all before your shift ends in the next hour. This is on your mind as you are taking Mrs. Jones’ vitals.


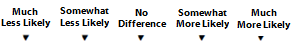
**Upon finishing taking her vitals, how much more/less likely are you to do hand hygiene upon exiting the room than in the scenario above (2a)?**

1. As you are leaving Mrs. Jones’ room, you take off the gloves you’ve been wearing.

***
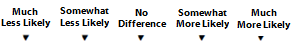
***

**How much more/less likely are you to do hand hygiene upon exiting the room than in the first scenario (2a)?**

1. As you are leaving Mrs. Jones’ room you notice that a fellow nurse is standing outside the doorway. You both make eye contact.

**
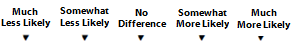
How much more/less likely are you to do hand hygiene upon exiting the room than in the first scenario (2a)?**

1. As you are leaving Mrs. Jones’ room you notice that the Infection Prevention director is standing outside the doorway.

**
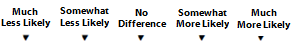
How much more/less likely are you to do hand hygiene upon exiting the room than in the first scenario (2a)?**

1. As you enter Mrs. Jones’ room, you see your nurse manager. The manager is talking with you and Mrs. Jones as you take Mrs. Jones’ vitals. Upon finishing, you and your nurse manager leave. The nurse manager does not practice hand hygiene.

**
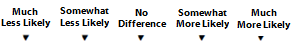
How much more/less likely are you to do hand hygiene upon exiting the room than in the first scenario (2a)?**

1. After taking Mrs. Jones’ vitals you say goodbye and turn to leave the room. You go to use the hand sanitizer dispenser in the room, but the dispenser is empty. The closest dispenser is down the hallway.

**
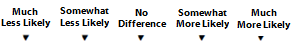
How much more/less likely are you to do hand hygiene upon exiting the room than in the first scenario (2a)?**

1. You have just finished taking Mrs. Jones’ vitals when another nurse urgently comes into the room and asks for your immediate assistance with a procedure in another room.

**
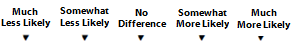
How much more/less likely are you to do hand hygiene upon exiting the room than in the first scenario (2a)?**

1. You are finishing taking Mrs. Jones’ vitals when there is a code. One of the patients on the floor is going in to cardiac arrest. You immediately respond to the code.

**
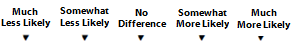
How much more/less likely are you to do hand hygiene upon exiting the room than in the first scenario (2a)?**

**Now instead of *exiting* Mrs. Jones’s room, you are ENTERING her room to take her vitals. Please answer the following questions.**

1. You need to take Mrs. Jones’ vitals. You have just been charting and responding to physician orders. You are in the hallway, about to go into Mrs. Jones’ room.

***
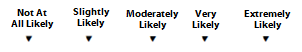
***

**How likely are you to practice hand hygiene upon entering the room?**

1. Upon entering Mrs. Jones’ room to take her vitals, she politely asks you to practice hand hygiene.

**
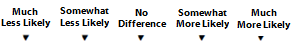
How much more/less likely are you to do hand hygiene upon entering the room than in the scenario above (2j)?**

1. You enter Mrs. Jones’ room and try to use the hand sanitizer, but the dispenser is empty. The closest dispenser is down the hallway.

**
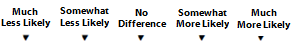
How much more/less likely are you to do hand hygiene upon entering the room than in the scenario 2j?**

1. Before entering Mrs. Jones’ room to take her vitals, you put on gloves.


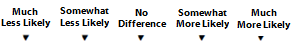
**How much more/less likely are you to do hand hygiene upon entering the room before putting your gloves on than in the scenario 2j?**

**SECTION C**

**Now we would like you to respond to a different situation. *Read the scenario and answer the following questions.***

**
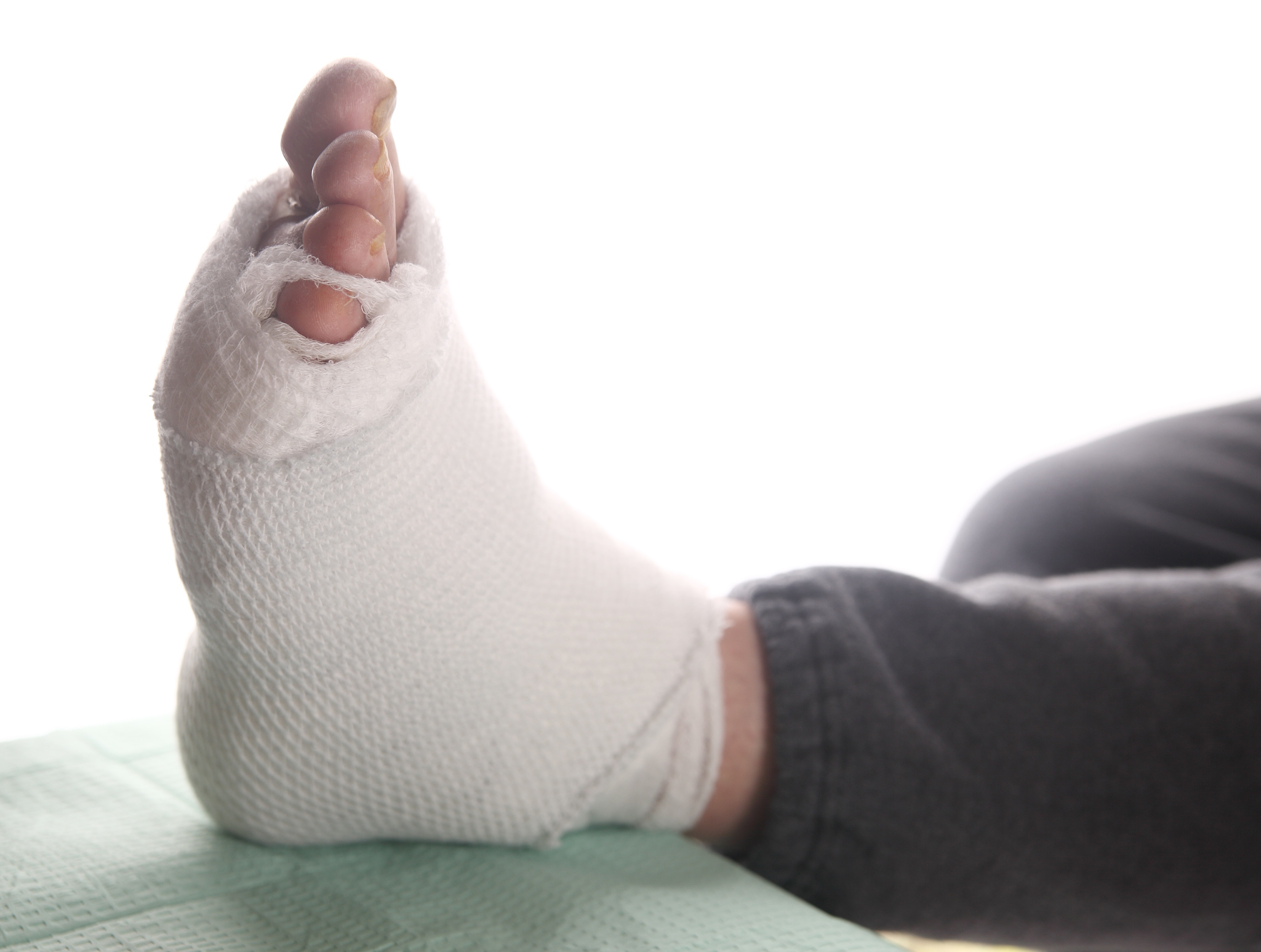
**

1. You are a nurse at Normal Hospital. You are cleaning and bandaging Mr. Robinson’s diabetic foot. After finishing the procedure, you take off your gloves, and then say goodbye to Mr. Robinson.

**
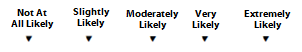
How likely are you to practice hand hygiene upon exiting the room?**

1. The unit is short-staffed today and you are busier than normal. You have to attend to two other patients, you need to debrief the attending physician, and fill out your shift report all before your shift ends in the next hour. This is on your mind as you finish cleaning and reapplying bandages to Mr. Robinson’s diabetic foot. Once again, you are wearing gloves.

**
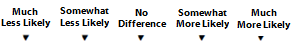
This time, how much more/less likely are you to do hand hygiene upon taking the gloves off and leaving the room than in the scenario above (3a)?**

1. You are leaving Mr. Robinson’s room you notice that a fellow nurse is standing outside the doorway. You both make eye contact.


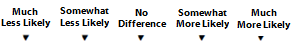
 **How much more/less likely are you to do hand hygiene upon taking the gloves off and exiting than in the scenario 3a?**

1. As you are leaving Mr. Robinson’s room you notice that the infection prevention director is standing outside the doorway.


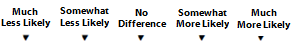
 **How much more/less likely are you to do hand hygiene upon taking the gloves off and exiting than in the first scenario (3a)?**

1. You enter Mr. Robinson’s room to clean and bandage his wound post-surgery and you see your nurse manager. The nurse manager talks with you and Mr. Robinson as you clean and apply bandages. Upon finishing, you and the nurse manager leave. The nurse manager does not practice hand hygiene.

**
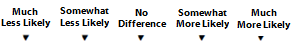
How much more/less likely are you to do hand hygiene upon exiting the room than in the first scenario (3a)?**

1. After cleaning and bandaging Mr. Robinson’s wound, you turn to leave the room. As you go to use the hand sanitizer dispenser in the room, you notice that it is empty. The closest dispenser is down the hallway.

**
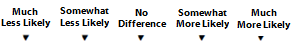
How much more/less likely are you to do hand hygiene upon exiting the room than in the first scenario (3a)?**

1. You have cleaned and bandaged Mr. Robinson’s wound when another nurse urgently comes into the room and asks for your immediate assistance. Once again, you are wearing gloves.

**
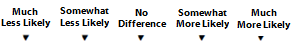
How much more/less likely are you to do hand hygiene upon exiting the room than in the first scenario (3a)?**

1. You have cleaned and bandaged Mr. Robinson’s wound and as you are about to leave his room there is a code. One of the patients on the floor is going in to cardiac arrest. You respond to the code. Once again, you are wearing gloves.

**
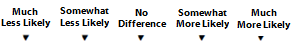
How much more/less likely are you to do hand hygiene upon exiting the room than in the first scenario (3a)?**

**Now instead of *exiting* Mr. Robinson’s room, you are ENTERING his room to clean and reapply his bandages. After reading each scenario, please answer the following questions.**

1. You need to clean and reapply bandages to Mr. Robinson’s wound. You have just been charting and responding to physician orders. You are in the hallways, about to go into Mr. Robinson’s room.

**
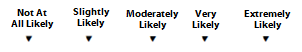
How likely are you to practice hand hygiene upon entering the room and putting on gloves?**

1. Upon entering the room, Mr. Robinson asks you politely to practice hand hygiene before putting on gloves.

**
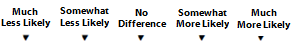
How much more/less likely are you to do hand hygiene upon entering the room and before putting on gloves than in the scenario above (3i)?**

1. You enter Mr. Robinson’s room and try to use the hand sanitizer before putting gloves on, but the dispenser is empty. The closest dispenser is down the hallway.

**
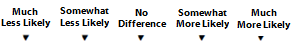
How much more/less likely are you to do hand hygiene upon entering the room and before putting on gloves than in the scenario above (3i)?**

**SECTION D**

***Please answer the following questions.***

1. **Which of these qualities or traits did you wish you had exhibited more of during your last shift? Choose *FIVE* from the following list.**

Empathy

Respect

Confidence

Technical Competence

Leadership

Good Communication Skills

Reliability

Awareness

Critical Thinking

Stress Management

Flexibility

Physical Endurance

Mental Endurance

Friendliness

Patience

Good Judgement

Patient Advocate

Resourcefulness

Responsiveness

Cooperativeness

1. **Which of the following statements would you LEAST like to hear said about you as a nurse? Choose *FIVE* from the following list.**

“You do not provide emotional support to your patients.”

“You are unsure of yourself as a nurse.”

“You do not handle stress well.”

“You are not as technically skilled as you should be.”

“You are curt and short with the patients.”

“You do not show leadership qualities.”

“You do not communicate well with others.”

“You neglected a patient.”

“You are not dependable.”

“You are not always aware of what is going on around you.”

“You hurt a patient.”

“You neglected a patient.”

“You do not know your patient’s wants or needs.”

“You are not flexible and able to adapt.”

“You are not a team player.”

**SECTION E**

***Please answer the following questions.***

**6. Out of 10 nurses working in your unit, how many do you think ALWAYS use hand sanitizer/soap:**

1. before entering a patient’s room? _____ out of 10
2. when exiting a patient’s room? _____ out of 10
3. after taking a patient’s vitals? _____ out of 10
4. after cleaning a patient’s wound? _____ out of 10
5. before charting in the nurse station? _____ out of 10
6. after talking to a colleague in the hallway? _____ out of 10

**
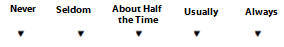
7. Do *YOU* think you SHOULD use hand sanitizer/soap:**

1. before entering a patient’s room? ...................
2. when exiting a patient’s room? ...................
3. after taking a patient’s vitals? ...................
4. after cleaning a patient’s wound? .................
5. before charting in the nurse station? .............

**
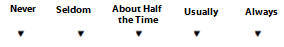
8. Do you believe most *OTHER NURSES* you work with THINK THAT YOU SHOULD use hand sanitizer/soap:**

1. before entering a patient’s room? ...................
2. when exiting a patient’s room? ...................
3. after taking a patient’s vitals? ...................
4. after cleaning a patient’s wound? ...................
5. before charting in the nurse station? ...................
6. after talking with fellow nurses in the ..................

break room?

1. **What would happen to a nurse in your unit if he/she did not practice appropriate hand hygiene? [open-ended]**

**10. If you saw a fellow nurse not use hand sanitizer/ soap after performing a procedure with a patient, what would you do? [open-ended]**

**SECTION F**

***Please indicate your agreement or disagreement with the following statements about hand hygiene.***

**14. Practicing hand hygiene before ENTERING a patient’s room is something:**

**
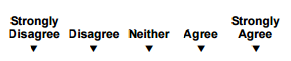
**

1. I do frequently. **....................................**
2. I do automatically. **....................................**
3. I do without having to **...............................**

consciously remember.

1. that makes me feel weird if I do not do it.
2. I do without thinking. **...............................**
3. that would require effort not to do it. **.........**
4. that belongs to my patient care routine. **.......**
5. I start doing before I realize I’m doing it. **....**
6. I would find hard not to do. **.......................**
7. I have no need to think about doing. **.........**
8. that’s typically “me.” **...............................**
9. I have been doing for a long time. **............**

**15. Practicing hand hygiene when EXITING a patient’s room is something:**

**
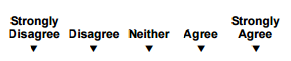
**

1. **
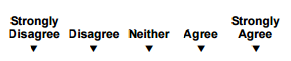
15a.** I do frequently. **.....................................**
2. I do automatically. **....................................**
3. I do without having to **...............................**

consciously remember.

1. that makes me feel weird if I do not do it.
2. I do without thinking. **...............................**
3. that would require effort not to do it. **.........**
4. that belongs to my patient care routine. **.......**
5. I start doing before I realize I’m doing it. **....**
6. I would find hard not to do. **.......................**
7. I have no need to think about doing. **.........**
8. that’s typically “me.” **...............................**
9. I have been doing for a long time. **............**

**Please rate your agreement with the following statements using the scale below.**

**
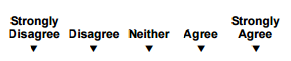
**

1. **A nurse’s practice of hand hygiene is influenced by their belief that fellow nurses think someone who doesn’t practice hand hygiene is a bad nurse.**
2. **Nurses should continue to practice hand hygiene consistently even if the administration and staff in their hospitals do not view it as a priority.**
3. **It is simply wrong NOT to practice appropriate hand hygiene.**
4. **Even if no one ever knew I was practicing hand hygiene, I would continue to do it every time.**
5. **I would rather be forced to wear flip-flops than not be allowed to do hand hygiene.**
6. **I would rather be forced to work 5 more hours a week at the same pay than not be allowed to do hand hygiene.**
7. **I would rather lose my lunch break than not be allowed to do hand hygiene.**

**SECTION G**

***Please read the stories below and respond to the following questions.***

A nurse uses hand sanitizer before entering a patient’s room. The nurse talks with the patient and asks about the patient’s pain levels and general mood. The nurse then takes the patient’s vitals and leaves the patient room. Upon exiting, the nurse uses hand sanitizer. The nurse manager has been watching this interaction and tells the nurse, “great job using the hand sanitizer! You are really on top of your game. Thanks for being responsible!”


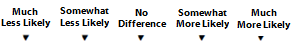


1. **If you were to receive this feedback, how much more likely are you to use hand sanitizer than you normally would do?**

A nurse uses hand sanitizer before entering a patient’s room. The nurse talks with the patient and asks about the patient’s pain levels and general mood. The nurse then takes the patient’s vitals and leaves the patient room. Upon exiting, the nurse uses hand sanitizer. The patient calls out to the nurse, “Thank you for using hand sanitizer. I feel safer and more confident in my care. I appreciate you doing that.”


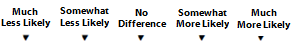


1. **If you were to receive this feedback, how much more likely are you to use hand sanitizer than you normally**

**would do?**

A nurse uses hand sanitizer before entering a patient’s room. The nurse talks with the patient and asks about the patient’s pain levels and general mood. The nurse then takes the patient’s vitals and leaves the patient room. Upon exiting, the nurse uses hand sanitizer. A fellow nurse has been watching this interaction and tells her colleague, “Thank you for using hand sanitizer. I feel safer and more confident in my care. I appreciate you doing that."


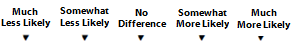


1. **If you were to receive this feedback, how much more likely are you to use hand sanitizer than you normally would do?**
2. **Was hand hygiene emphasized during your training as a nurse?**

Yes  No

1. **Have you taken a continuing education course on hand hygiene?**

Yes  No

1. **Have there been any hand hygiene initiatives or programs at the facility where you work?**

Yes  No

1. **Is hand hygiene regarded as a priority at the facility where you work?**

Yes  No

1. **Are nursing staff in your unit ever punished (even if only verbally) for not practicing hand hygiene?**

Yes  No

1. **Are nursing staff in your unit ever rewarded (even if only verbally) for properly practicing hand hygiene?**

Yes  No

**Please indicate your agreement or disagreement with the following statements about your “unit.”**

***NOTE: “Patient safety”*** *is defined as the avoidance and prevention of patient injuries or adverse events resulting from the processes of health care delivery.*

*
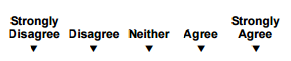
*

1. **Hospital management provides**

**a work climate that promotes**

**patient safety.**

1. **Patient safety is never sacrificed**

**to get more work done.**

1. **Our procedures and systems are**

**good at preventing errors**

**from happening.**

1. **Staff feel like their mistakes are**

**held against them.**

1. **Nurses in our unit help each**

**other out regularly.**

1. **I can depend on getting help from**

**other nurses.**

1. **In this unit, people treat each other**

**with respect.**

1. **Some of my closest friends are my**

**work colleagues.**

1. **We sometimes work in “crisis mode”**

**trying to do too much, too quickly.**

1. **Hand hygiene is considered to be a part**

**of patient safety where I work.**

1. **My supervisor/manager overlooks**

**patient safety problems that**

**repeatedly happen.**

1. **My supervisor/manager seriously**

**considers staff suggestions for**

**improving patient safety.**

1. **My supervisor/manager says**

**a good word when he/she sees a job**

**done according to established**

**patient safety procedures.**

1. **Hospital management seems interested in**

**patient safety only after an adverse event**

**happens.**

**How often do the following things happen in your work area/unit?**

**
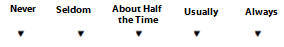
**

1. **Staff will freely speak up if they**

**see something that may negatively**

**affect patient care.**

1. **In this unit, we discuss ways to**

**prevent errors from happening again.**

1. **Staff feel free to question the decisions or**

**actions of those with more authority.**

1. **Staff are afraid to ask questions when**

**something does not seem right.**

1. **When a mistake is made that**

***could harm the patient*, but does not,**

**how often is this reported?**

1. **What is your job title?**

Staff Nurse

Nurse Manager

Assistant Nurse Manager

Nursing Director

Clinical Educator

Advanced Practice Nurse

Other:

1. **Most of my patients in my current job are:**

Adults

Paediatric

Neonatal

Multiple Age Groups

I do not provide direct patient care

1. In a typical work week, about what percent of your work time is spent performing the following kinds of tasks?

Note: Your responses should add up to 100%.

_____% **Interacting with patients** (All nurse/patient time, either in the patient’s room or in the hallways, e.g., transporting, ambulating)

_____% **Preparing therapies** (Preparing intravenous therapies, medications, treatments, etc.)

_____% **Shift change activities** (Reporting, counting narcotics, getting assignments, and making patient rounds, either during or after report)

_____% **Professional interaction** (All face-to-face communications except communications with patients. For example, communications with visitors, doctors, social workers, other nurses, hospital staff.)

_____% **Paperwork** (Charting, checking physician orders, filling out forms, incident reports, requisitions, or any other paperwork)

_____**% Phone/E-mail communications**

_____% **Obtaining supplies** (time spent outside of the patient’s room looking for or obtaining supplies, but not requisitioning)

_____% **Other** (Any activity not listed above, including meals and break time)
