# Supplementary material for: Determinants of hand hygiene compliance among nurses in US hospitals: A formative research study
Source: PLoS One. 2020 Apr 7;15(4):e0230573. doi: 10.1371/journal.pone.0230573 (PMC7138309; doi:10.1371/journal.pone.0230573)
Supplement: S1 Fig — (DOCX) [file pone.0230573.s003.docx]

**Supplement 3- Figure A: Comparison of Base Vignettes.** This graph compares the Likert-scale responses for the exit and entry scenarios of the vitals vignette and for the exit and entry scenarios of the diabetic foot wound vignette.

**Supplement 3- Figure B: Factors for the Vitals Vignette Exit.** This graph displays the responses for each of the factors considered in the vitals vignette upon exiting a patient’s room.

**Supplement 3- Figure C: Factors for the Vitals Vignette Entry.** This graph displays the responses for each of the factors considered in the vitals vignette upon entering a patient’s room.

**Supplement 3- Figure D: Factors for the Cleaning Wound Vignette Exit.** This graph displays the responses for each of the factors considered in the wound vignette upon exiting a patient’s room.

**Supplement 3- Figure E: Factors for the Cleaning Wound Vignette Entry**. This graph displayed the responses for each of the factors considered in the wound vignette upon entering a patient’s room.

**Supplement 3- Figure F: Role— Top Five Most Desirable Traits and Qualities.** Respondents were asked to choose five qualities or traits that they wished they had exhibited more of during their last shift.

**Supplement 3- Figure G: Role— Top Five Negative Statements.** Respondents were asked to choose five statements that they would least like to hear said about them as a nurse.

Supplement 3- Figure H: Comparison of Mean Responses for Empirical Expectations Question.

**Supplement 3- Figure I: Normative Personal Beliefs.** Respondents were asked if they thought they should practice hand hygiene at various moments.

**Supplement 3- Figure J: Normative Expectations.** Respondents were asked if they beliefs that most other nurses thought they should practice hand hygiene at various moments.

Supplement 3- Figure K: Motivation and Feedback.
